# Multidisciplinary team management and the survival of head and neck cancer patients: a systematic review and meta-analysis

**DOI:** 10.1097/MS9.0000000000005479

**Published:** 2026-09-18

**Authors:** Hanan M. Qasem, Ali O. Aldamen, Muhammad Idrees, Ragad Ala’a Tawalbeh, Mohammed Fawaz Alqudah, Ja’Far Mohamad Matalgah, Sakhr Alshwayyat, Ayham Mohammad Hussein, Haya B. Abukharmh, Mohammed Al-mahdi Al-kurdi

**Affiliations:** a Faculty of Dentistry, Jordan University of Science and Technology (JUST), Irbid, Jordan; b Faculty of Medicine, Al Yarmouk University, Irbid, Jordan; c Department of Internal Medicine, Lahore General Hospital, Punjab, Pakistan; d Faculty of Medicine, Jordan University of Science and Technology, Irbid, Jordan; e Faculty of Medicine, Al-Balqa’ Applied University, Salt, Jordan; f Faculty of Pharmacy, Yarmouk University, Irbid, Jordan; g Faculty of Medicine, University of Aleppo, Aleppo, Syria

**Keywords:** head and neck cancer, MDT, multidisciplinary team, survival

## Abstract

**Introduction:**

Multidisciplinary teams (MDTs) in cancer care aim to improve patient outcomes by bringing together healthcare professionals from various specialties. This collaborative approach has been associated with improved decision-making and survival rates, particularly in the management of complex cases, such as head and neck cancer (HNC). Our study aimed to assess the impact of MDTs on overall survival (OS) in patients at different stages of HNC compared with those who received non-MDT care.

**Methods:**

A systematic search was performed following PRISMA guidelines in PubMed, Scopus, Embase, and Web of Science databases from inception to June 2024 for studies comparing MDT management of patients with HNC with conventional treatment. The review was registered in PROSPERO under the number CRD420251165637. Data analysis was conducted using Review Manager version 5.4.

**Results:**

A systematic review and meta-analysis were conducted, including nine studies with a total of 42 937 patients. The pooled analysis showed that MDT management was associated with a significant 13% improvement in survival rates for HNC patients (HR: 0.87, 95% CI: 0.78–0.97, *P* = 0.02). For patients with stage IV HNC, MDT care was not associated with a statistically significant survival improvement; however, this subgroup analysis included only three studies and should be interpreted cautiously (HR: 0.80, 95% CI: 0.62–1.03, *P* = 0.09). Quality assessment of the included studies indicated that most were of good quality, though one study was rated as fair due to methodological limitations.

**Conclusion:**

MDTs can improve survival rates among patients with HNC. Their effect in stage IV disease remains inconclusive. Further research is needed to explore the economic impact of MDTs and their effectiveness in broader populations and advanced stages.

## Graphical Abstract

**Figure:**
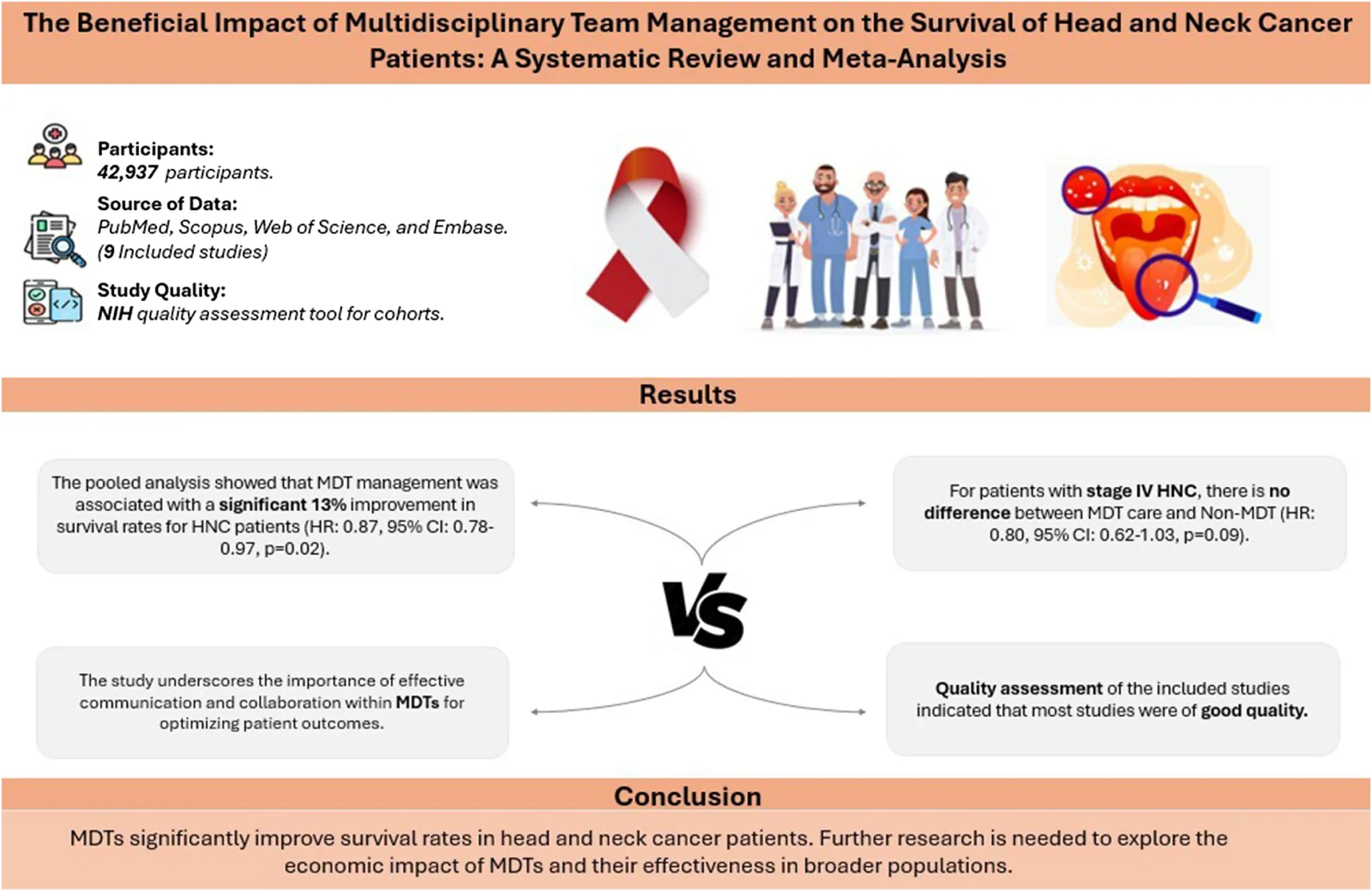

## Introduction

Multidisciplinary teams (MDTs) in cancer care comprise healthcare professionals from various specialties who collaborate to deliver comprehensive patient care. These teams typically include oncologists, surgeons, radiologists, pathologists, and allied health professionals, each providing specialized expertise for individualized treatment plans. The primary goal of MDTs is to enhance decision-making quality and improve patient outcomes by consistently applying evidence-based practices, resulting in a 25% improvement in survival rates and a 20% reduction in treatment errors^[^1^]^. However, status hierarchies within teams can affect decision-making, with 60% of members perceiving hierarchical barriers impacting discussions^[^2^]^, underscoring the need for inclusive leadership and effective communication to optimize performance.

HIGHLIGHTS

- This systematic review and meta-analysis consolidates data from over 42 000 patients with head and neck cancer to evaluate the effect of multidisciplinary team (MDT) management on survival outcomes.
- MDT care improves overall survival by approximately 13%, emphasizing the tangible clinical value of collaborative decision-making in complex oncologic cases.
- The analysis underscores that MDT benefits are most pronounced in early and intermediate cancer stages, whereas the effect in stage IV disease remains less definitive.
- Integrating diverse clinical expertise within MDTs enhances treatment precision, reduces therapeutic delays, and aligns care with evidence-based protocols across healthcare systems.
- Findings advocate for broader institutional adoption of structured MDT frameworks, encouraging health policy reforms to support multidisciplinary collaboration in oncology worldwide.

Research indicates that MDTs improve treatment decision-making by 35% and streamline care processes, leading to more effective and personalized pathways and a 30% reduction in treatment delays^[^3^]^. MDT meetings facilitate comprehensive strategies that consider multiple perspectives, aiding the holistic management of complex cancer cases and improving patient outcomes by up to 40%^[^4^]^. Additionally, embedding oncology care coordination within broader support programs enhances interdisciplinary collaboration, addresses unmet needs, reduces healthcare utilization by 20%, and improves patient satisfaction by 15%^[^5^]^. This collaborative approach supports continuous care from diagnosis through survivorship, ensuring comprehensive and tailored care, resulting in a 25% improvement in quality of life and better health outcomes^[^6^]^.

A study on non-small cell lung cancer showed that patients managed by MDTs had a 20% increase in overall survival (OS) rates and a 15% reduction in time to treatment compared with those not managed by such teams^[^7^]^. A scoping review protocol identified barriers in oncology management, such as communication issues and hierarchical dynamics, providing a framework for future systematic reviews aimed at improving patient outcomes by 10%^[^8^]^. Recent healthcare research has also explored digital and artificial-intelligence-based tools as supportive resources for clinical decision support and health management. Guo *et al* reported that ChatGPT-related healthcare research includes clinical decision support, patient education, information integration, and scientific writing, but also highlighted unresolved concerns regarding reliability, interpretability, privacy, and responsibility. Although these tools may support future information organization within complex care pathways, MDT-based HNC management remains dependent on expert multidisciplinary clinical judgment^[^9^]^.

Although previous meta-analyses have evaluated the association between MDT care and survival in HNC, it is still unclear whether MDT care improves survival in advanced cancer stages. The present review provides an updated synthesis by including a larger patient population, more recent studies, and broader geographic representation. In addition, we performed subgroup analyses according to tumor type, country, and stage IV disease, thereby providing further insight into the contexts in which MDT care may be most strongly associated with improved survival. Therefore, our study aims to assess the impact of the MDT approach on the OS of patients with different stages of cancer compared with patients receiving non-MDT conventional treatment. It focuses on the association between MDT care and OS in HNC across stages, with special attention to stage IV disease, where treatment decisions are most complex and the available evidence remains limited, and it provides concrete evidence on the effectiveness of MDTs, potentially guiding future clinical practices and healthcare policies.

## Methods

This systematic review and meta-analysis was reported in line with the PRISMA (Preferred Reporting Items for Systematic Reviews and Meta-Analyses) guidelines^[^10^]^. This systematic review and meta-analysis was registered in the International Prospective Register of Systematic Reviews (PROSPERO) under registration number CRD420251165637. This manuscript was also prepared in accordance with the AMSTAR and TITAN guidelines^[^11,12^]^.

### Search strategy and eligibility criteria

A comprehensive and systematic search was conducted in the main databases: Web of Science, Embase, Scopus, and PubMed. The keywords used for the search strategy were extracted from Medical Subject Headings (MeSH). The search strategy was built using a combination of keywords related to MDT and HNC, such as: “Multidisciplinary team,” “MDT,” “Head and Neck cancer,” “Oral cancer,” “survival,” “Prognosis,” and “HNC.” More details are provided in Supplemental Digital Content Table S1, available at: https://links.lww.com/MS9/B342.

The studies included in our paper adhere to the following criteria: (1) human-based research conducted in English, (2) MDT management of patients, (3) focus on head and neck cancers, (4) measurement of OS of participants, and (5) inclusion of only published articles. The following studies were excluded: (1) abstracts without published full texts, (2) review or editorial articles, (3) studies with a single-discipline management approach, and (4) articles not published in English. After removing duplicates, two reviewers independently assessed the titles and abstracts of studies imported from the database, excluding those that did not meet our inclusion criteria. Full-text screening was then conducted, and any discrepancies were resolved through consensus and consultation with a supervisor.

### Data extraction

Two independent authors extracted the data using a standardized Excel spreadsheet, which included the following information: authors' names, publication year, study design, country, number of participants, mean age, follow-up duration, study aim, and conclusion. Any discrepancies were resolved by consensus, with a third investigator involved if necessary. In addition, the operational definitions and composition of MDT care were extracted from each included study (Table 1 and Table 2).

**Table 1:** Characteristics of included studies.

Study	Year	Number of patients	Age	Country	Period	Aim	Conclusion
Hamilton *et al*^[^13^]^	2013	209	70.2	England	1996–2010	The Cumberland Infirmary, Carlisle, serves a largely remote, rural population of 330 000. The aim of this study was to report the treatment and survival figures for patients treated for laryngeal cancer at this center.	Our tumor-specific survival rate was very high, and this success may be due in part to high rates of surgical intervention. Survival data compared favorably with other centers, despite less radical radiotherapy regimes. Laryngeal cancer can be managed effectively in a small, relatively remote, multidisciplinary team (MDT) setting.
Liao *et al*^[^14^]^	2016	1616	51	Taiwan	1996–2011	The purpose of this study was to investigate whether MDT care (MDTC) is associated with outcomes in oral cavity squamous cell carcinoma (SCC).	The association of MDTC with improved outcomes may be potentially explained by a better therapeutic alliance between the patient and the tumor board, and/or a greater thoroughness in clinical management
Light *et al*^[^15^]^	2017	106	62	America	2003–2011	Treatment of head and neck cancer is complex, and a multidisciplinary clinic may improve the coordination of care.	Implementation of a multidisciplinary clinic improved care coordination and disease-specific survival in patients with oropharyngeal SCC.
						The value of a head and neck multidisciplinary clinic has not yet been established in oropharyngeal squamous cell carcinoma (SCC).	
Friedland *et al*^[^16^]^	2011	726	-Not reported	Australia	1996–2008	We analyzed the outcomes of 726 cases of primary head and neck cancer patients managed between 1996 and 2008, including those managed in the multidisciplinary clinic or team setting (MDT) and those managed outside of an MDT by individual disciplines (non-MDT) in the same institution.	The improved survival of MDT-managed stage IV patients probably represents both the selection of multimodality treatment and chemotherapeutic advances that these patients received in a MDT setting by head and neck cancer specialists as opposed to cancer generalists in a non-MDT setting.
Liu *et al*^[^17^]^	2020	771	62	Australia	2008–2016	Oral SCC has been traditionally described as a highly lethal disease. This study aims to provide updated multi-institutional data on the survival of oral SCC patients in Australia	Survival outcomes for oral SCC among Australian patients have improved, possibly due to advances in multidisciplinary care. Early detection of oral SCC leads to highly favorable prognosis; there is therefore an opportunity for routine oral cancer screening to be performed by community health practitioners with the aim of improving population survival from oral SCC.
Meltzer *et* *al*^[^18^]^	2021	3081	64.3	America	2009–2017	To compare survival among patients with head and neck cancer before and after implementing a weekly multidisciplinary clinic and case conference	Among 3081 patients diagnosed with HNC at multiple tumor sites, MDC and case conference implementation was associated with improved outcomes, particularly for advanced stage tumors, that were related to more consistent use of guideline directed surgical and nonsurgical treatment. We found evidence of differential impact across tumor sites, but this finding may have been observed by chance and warrants confirmation in larger populations with contemporaneous control groups. The findings offer further evidence that MDC is most beneficial for patients with advanced disease, affirming the value of resource- and time-intensive MDT management to improved survival.
Tsai *et* *al*^[^19^]^	2014	16 691	53.6	Taiwan	2005–2010	The aim of this study was to investigate the association between MDT management and survival of oral cavity cancer patients using a nationwide database in Taiwan.	Survival rates in oral cavity cancer patients with MDT management appeared to be marginally better than those of patients without MDT management
Liu *et* *al*^[^20^]^	2019	224	61.4	America	2006–2015	Although the multidisciplinary tumor board (MTB) is accepted as best practice for the management of head and neck SCC (HNSCC), there is limited evidence showing its impact on survival. Our goal was to investigate the impact of an MTB following the hiring of a fellowship-trained head and neck surgeon and implementation of an MTB at our institution. We hypothesized that these changes would demonstrate an improvement in survival.	Our study demonstrates that treatment of HNSCC by a dedicated MDT results in improved survival. Multidisciplinary care should be considered best practice in the care of HNSCC.
Wang *et al*^[^19^]^	2012	19,513	53.29	Taiwan	2004–2007	The incidence of oral cavity cancer is high in Taiwan. To improve patient survival, MDT care was implemented. This research compared the survival of MDT care participants/ non-participants and examined the effect of MDT care on patient characteristics	The relative risk of death was lower for MDT care participants. The effect of MDT care was stronger among older patients and patients with fewer comorbidities.

**Table 2:** Definitions and characteristics of MDT management across included studies.

Study ID	MDT definition/description used in study	MDT members included	MDT function/approach
Hamilton *et al*, 2013^[^13^]^	The study described MDT as a “head and neck multidisciplinary team (MDT)” based at Cumberland Infirmary that managed and rehabilitated patients with laryngeal cancer in a small rural cancer center. MDT care involved coordinated management of diagnosis, treatment planning, treatment delivery, rehabilitation, and clinical data verification.	Explicitly mentioned members included: head and neck surgeon, oncologist, and MDT coordinator. The study also referred to a “specialist head and neck MDT,” implying involvement of specialized head and neck cancer care professionals.	The MDT collaboratively managed patient care throughout diagnosis, treatment, rehabilitation.
Liao *et al*, 2016^[^14^]^	MDT was defined as a formal “multidisciplinary team care (MDTC)” approach introduced in 2004 for comprehensive management of oral cavity SCC. The study emphasized integrated and individualized patient management through weekly MDT tumor board meetings and coordinated case management.	Explicitly included: surgeons, radiation oncologists, medical oncologists, pathologists, radiologists, nuclear medicine physicians, dentists, supportive care providers, psychologists, nurses, nutritionists, social workers, and case managers.	MDT functioned through weekly tumor board meetings in which all diagnostic and treatment options were discussed to develop individualized treatment plans. Case managers supervised treatment course and follow-up schedules,
Light *et al*, 2017^[^15^]^	The study defined MDT as a “multidisciplinary head and neck cancer clinic” established to minimize complexities in care coordination for patients with oropharyngeal SCC. MDT care focused on integrated, team-based management and face-to-face collaboration among clinicians for treatment planning and supportive care.	Explicitly included: head and neck surgeons, radiation oncologists, medical oncologists, speech-language pathologists, nutritionists/dietitians, social workers/cancer care navigators, palliative care physicians, dentists, and supportive care specialists.	MDT functioned through a dedicated multidisciplinary clinic where patients were evaluated by multiple specialists in a coordinated setting. The approach aimed to streamline treatment pathways, improve communication among clinicians, reduce treatment delays, coordinate supportive services, and optimize treatment and functional outcomes.
Friedland *et al*, 2011^[^16^]^	The study described MDT as a “multidisciplinary clinic or team setting” where “medical and allied health specialties are represented so that accurate tumor staging and treatment plans can be best tailored to individuals.” The MDT reviewed “each new patient’s diagnosis, imaging, medical and social factors, confirmed staging and formulated a management plan.”	Otolaryngologists, plastic surgeons, radiation oncologists, medical oncologists, dentists, dieticians, speech pathologists, radiologists, and a nurse cancer care coordinator.	Weekly head and neck cancer MDT meeting at Sir Charles Gairdner Hospital. Each new patient was reviewed by the MDT, and cases were re-discussed whenever further treatment was planned. The MDT followed National Comprehensive Cancer Network (NCCN) Clinical Practice Guidelines in Oncology.
Tsai *et al*, 2015^[^19^]^	MDT care was described as an approach that “improves upon conventional managements for oral cavity cancer” through integration of multiple specialties to improve patient management and quality of life. The study specifically stated that, in Taiwan, MDT management for oral cavity cancer required representation of all medical/surgical specialties and routine combined conferences to discuss newly diagnosed patients	Mandatory MDT members included: head and neck surgeon, radiation oncologist, medical oncologist, pathologist, and radiologist. The study additionally described MDT care as integrating surgeons, radiation oncologists, medical oncologists, psychologists, dietitians, speech therapists, and nursing staff.	MDT management was part of a national healthcare policy implemented by Taiwan’s National Health Insurance Administration in 2003. Hospitals were required to conduct routine combined conferences for newly diagnosed oral cavity cancer patients, and institutions received additional reimbursement for properly documented MDT-managed cases.
Wang *et al*, 2012^[^21^]^	MDT care was defined as an approach that “goes beyond conventional treatments for oral cavity cancer, such as surgical excision, radiation therapy, and chemotherapy.” The study described MDT care as a comprehensive cancer treatment model integrating multiple disciplines to improve quality of care and survival outcomes.	The MDT was described as integrating surgeons, tumor physicians/medical oncologists, radiology physicians, psychologists, physiatrists, and dietitians.	MDT care was implemented nationally in Taiwan since April 2003 through the Taiwan Bureau of National Health Insurance as part of a formal “multidisciplinary team care for cancer patients” policy intended to provide a complete cancer treatment scheme. Patients were categorized according to participation versus non-participation in MDT care.
Liu *et al*, 2019^[^20^]^	MDT was defined as a formal weekly multidisciplinary tumor board in which all new head and neck cancer cases were reviewed for collaborative treatment planning and ongoing coordinated care.	Core members: fellowship-trained head and neck surgeon, medical oncology, radiation oncology. Additional members: neuroradiology, speech therapy/speech-language pathology, nutrition, pathology, dental services, and social work. Before 2011, the team consisted of non-fellowship-trained otolaryngologists, medical oncologists, and radiation oncologists.	Weekly formal conferences; all new cases discussed; patients receiving surgery, radiation, and/or chemotherapy were also reviewed weekly during treatment to coordinate specialty, ancillary, and social services.
Meltzer *et al*, 2021^[^18^]^	MDT was defined as a centralized weekly multidisciplinary clinic (MDC) and virtual case conference in which all newly diagnosed and recurrent head and neck cancer patients were referred for multidisciplinary evaluation, staging review, treatment planning, and coordinated management according to NCCN guidelines.	Fellowship-trained head and neck surgeons, radiation oncologists, medical oncologists, speech and language pathologists, dietician’s, social workers, neuroradiologists, nuclear medicine specialists, and pathologists.	Patients were evaluated at 3 regional HNC hubs, followed by weekly virtual multidisciplinary conferences where imaging, pathology, staging, and treatment plans were reviewed and modified collaboratively.
Liu *et al*, 2020^[^17^]^	MDT was defined as head and neck MDT meetings in which patients with newly diagnosed oral SCC were discussed after staging investigations, before initial treatment, and again after treatment to determine adjuvant therapy and assess treatment response.	Surgeons, radiation oncologists, medical oncologists, radiologists, pathologists, and allied health professionals.	MDT meetings were used for collaborative staging review, treatment, discussion, adjuvant therapy decisions.

### Quality assessment

We used the NIH tool to assess the quality of the nine studies included in our systematic review and meta-analysis. This tool comprised 14 criteria that evaluated key aspects of study methods and implementation, such as the research question, study population, exposure measurement, outcome measurement, confounding adjustment, and statistical analysis. Each criterion was rated as “yes” (1 point), “no” (0 points), or “other.” The total score for each study was calculated by summing the number of “yes” ratings, with a maximum possible score of 14. Studies scoring 10 or higher were classified as good quality; those scoring between 5 and 9 as fair quality; and those scoring 4 or lower as poor quality.

### Data analysis

Statistical analysis was conducted using Review Manager version 5.4 software (The Cochrane Collaboration, Copenhagen, Denmark). We presented the pooled results by the generic inverse variance method with 95% CIs and hazard ratios (HRs), and the Mantel–Haenszel method. We used a random-effects model for all analyses. Statistical heterogeneity was tested using the chi-square test and the *I*^2^ statistic. We did not evaluate the risk of publication bias by funnel plot asymmetry testing or Egger’s test because the number of eligible studies for individual results did not exceed ten. Sensitivity analysis was performed by leaving one study out at a time to explore whether any individual study had a major influence on the pooled estimate or statistical heterogeneity. These analyses were exploratory and did not replace the primary pooled analysis. Studies that did not provide extractable data for a specific outcome or subgroup were not included in that specific meta-analysis but remained part of the qualitative synthesis.

## Results

### Search results

Our search strategy identified 962 studies from the MEDLINE (via PubMed), Web of Science, Scopus, and Embase databases. After eliminating duplicates, 503 studies were screened by title and abstract, leading to the exclusion of 494 studies. The remaining nine full-text articles were independently assessed by the same authors and were all considered eligible for inclusion in the qualitative and quantitative analyses^[^13–20,20^]^. The search and selection procedures are depicted in Figure 1.

**Figure 1.**
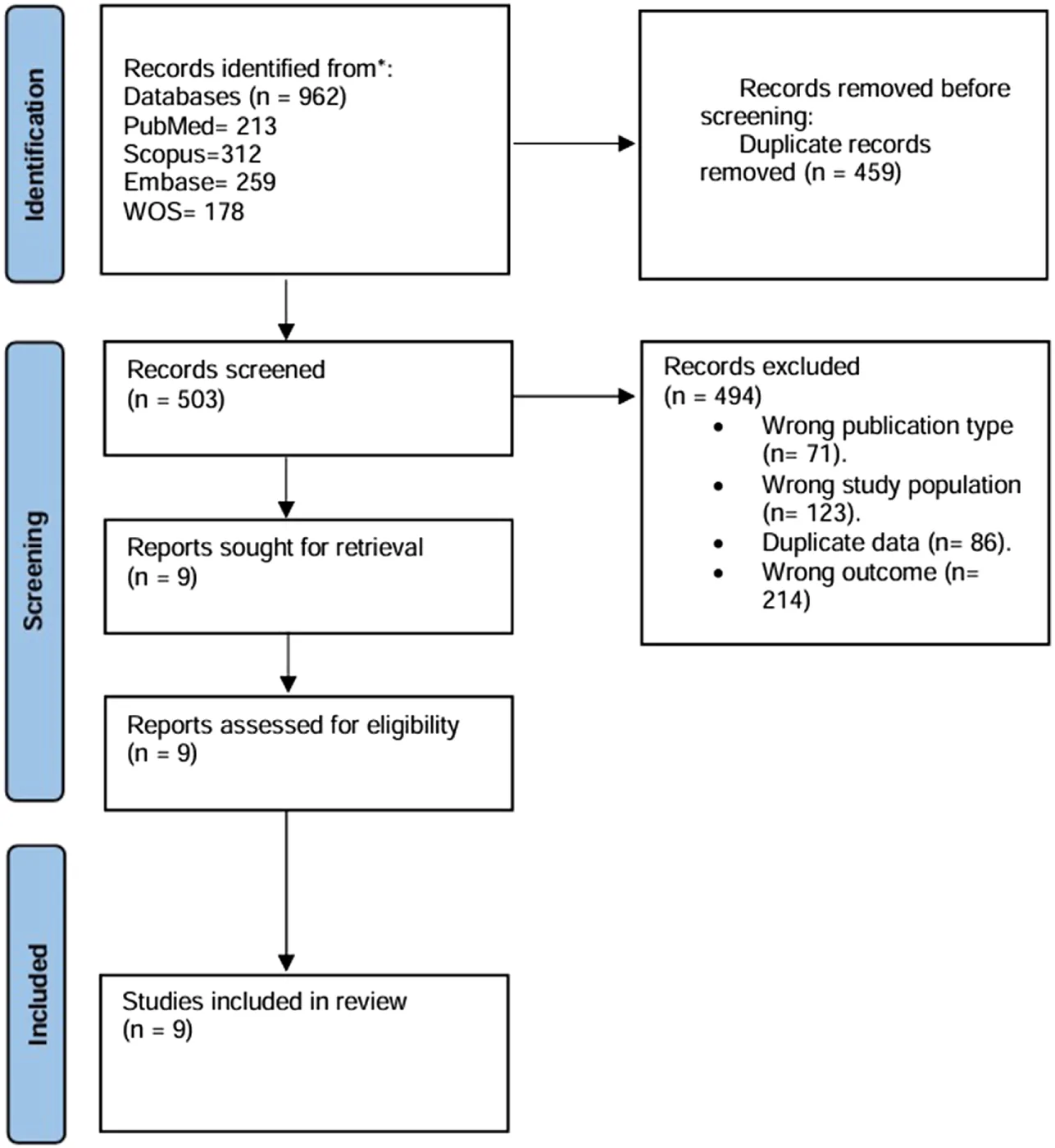
Flow chart of the selection process.

### Summary of included studies

Nine studies were included in our review. In total, 42 937 patients were included in our analysis, with the mean age ranging from 51 to 70.2 years. The number of studies, subgrouped by country, was as follows: three from Taiwan, two from Australia, three from the United States, and one from England. The publication period was between 2011 and 2021. However, the patient recruitment period was between 1996 and 2017. The characteristics of the included studies are summarized in Table 1. MDTs commonly include head and neck surgeons, medical oncologists, radiation oncologists, radiologists, pathologists, specialist nurses, speech and swallowing therapists, dietitians, dental/maxillofacial specialists, and palliative/supportive care professionals when appropriate. Additionally, patient attendance at MDT meetings was not consistently reported across the included studies (Table 2).

### Outcomes

#### The effect of MDT on the survival rate of patients with HNC

Seven studies were included in this analysis. A statistically significant difference in the survival rate of patients with HNC between the MDT and non-MDT groups was observed [pooled HR: 0.87, 95% CI (0.78–0.97), *P* = 0.02; Fig. 2]. MDT is significantly associated with a 13% improvement in survival rate. The heterogeneity was high (*I*^2^ = 75%, *P* = 0.0005). When we removed the study by Meltzer *et al*, the heterogeneity dropped to 61%. Subgroup analysis by type of tumor was performed (Supplemental Digital Content Figure S1, available at: https://links.lww.com/MS9/B342 However, there was no statistical difference between the groups (*P* = 0.39; Supplemental Digital Content Figure S2, available at: https://links.lww.com/MS9/B342). Subgroup analysis by country revealed a significant impact of MDT care in Australia [pooled HR: 0.79, 95% CI (0.64–0.98), *P* = 0.03] and Taiwan [pooled HR: 0.86, 95% CI (0.77–0.96), *P* = 0.007]. However, there was no difference between groups in the United States [pooled HR: 0.87, 95% CI (0.55–1.37), *P* = 0.55; Supplemental Digital Content Figure S3, available at: https://links.lww.com/MS9/B342].

**Figure 2.**
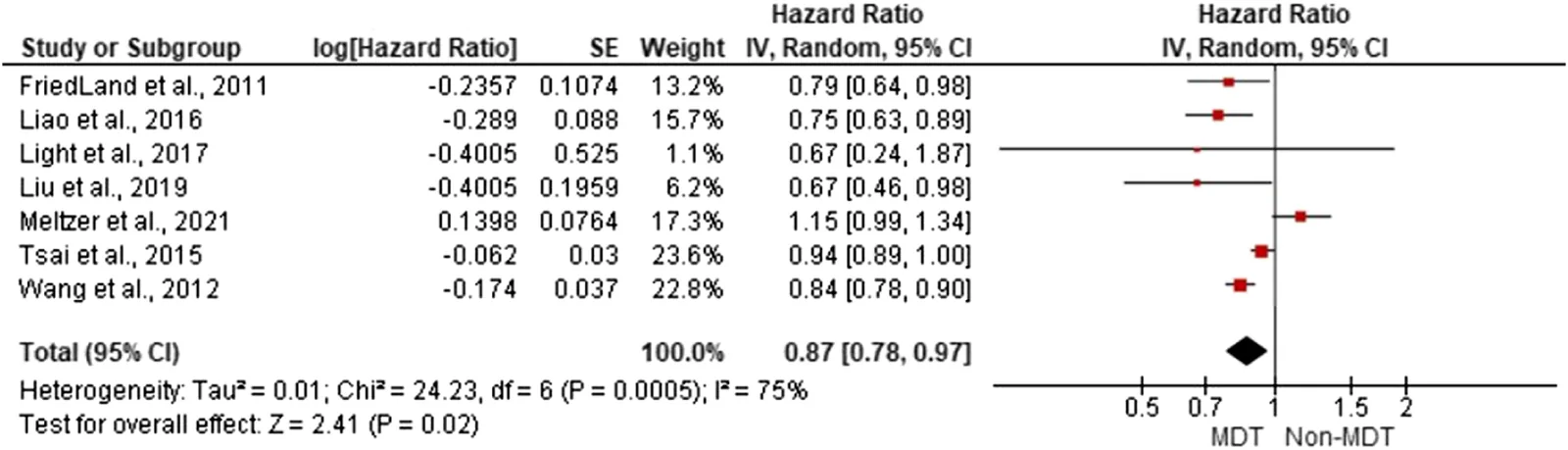
Forest plot of the survival rate of MDT patients.

#### The effect of MDT on the survival rate of patients with stage IV HNC

Three studies were included in this analysis. A statistically insignificant difference in the survival rate of patients with HNC between the MDT and non-MDT groups was observed [pooled HR, 0.80; 95% CI (0.62–1.03), *P* = 0.09; Fig. 3]. The heterogeneity was high (*I*^2^ = 62%; *P* = 0.06). When we removed the study by Friedland *et al*, the heterogeneity dropped to 0%. Subgroup analysis by tumor type was performed; however, there was no statistical difference between the groups (*P* = 0.06; Supplemental Digital Content Figure S4, available at: https://links.lww.com/MS9/B342). Subgroup analysis by country was performed, and there was no statistical difference between the groups (*P* = 0.25; Supplemental Digital Content Figure S5, available at: https://links.lww.com/MS9/B342). However, because this analysis included only three studies and demonstrated heterogeneity, the finding should be interpreted cautiously and considered a suggestion rather than a definitive conclusion.

**Figure 3.**
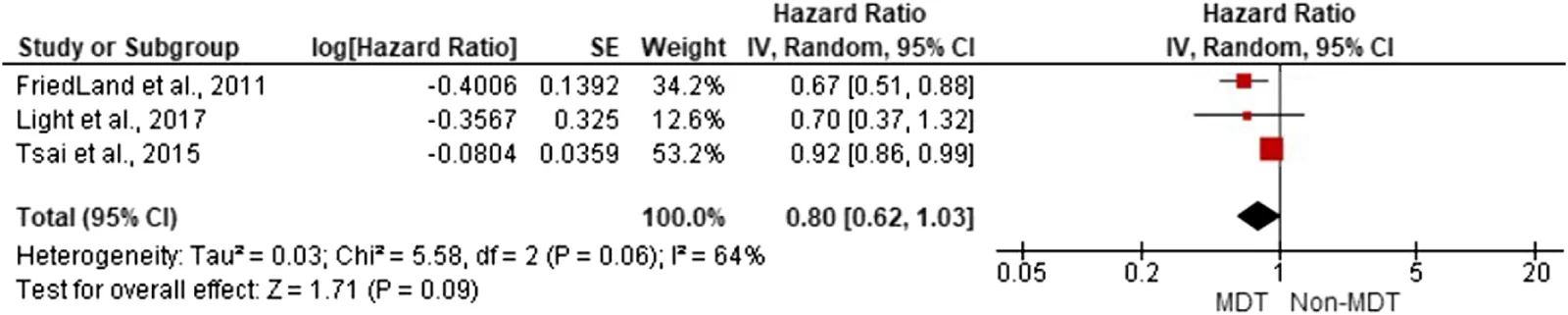
Forest plot of the MDT survival rate in patients with stage IV cancer.

### Quality assessment

We conducted a quality assessment of nine retrospective cohort studies. Using the NIH Study Quality Assessment Tool as a reference, eight studies were classified as good quality, while one study was rated as fair quality. The main methodological limitations were related to the absence of sample size justification or power calculation, lack of repeated exposure assessment over time, unclear or absent blinding of outcome assessors, and incomplete reporting of loss to follow-up in several studies. These limitations were considered when interpreting the certainty of the evidence. Further details of the quality assessment are provided in Supplemental Digital Content Table S2, available at: https://links.lww.com/MS9/B342.

## Discussion

This systematic review and meta-analysis provides an updated synthesis of the available evidence on the association between MDT management and OS in patients with head and neck cancer (HNC). A key strength of this review is that it included nine studies with a total of 42 937 patients, providing a broad summary of the available comparative evidence. In addition, the review followed PRISMA guidelines, searched four major databases, and assessed study quality using the NIH Study Quality Assessment Tool. The analysis showed that MDT management was associated with improved OS in patients with HNC, with a pooled HR of 0.87, corresponding to a 13% relative reduction in the hazard of death compared with non-MDT or conventional management.

The potential benefit of MDT care should not be interpreted as suggesting that major treatment decision errors are common in conventional care, particularly because evidence-based guidelines for HNC management are well established. Rather, MDTs may improve the individualized application of these recommendations in complex clinical scenarios requiring multimodality treatment and supportive care coordination. The value of MDT care may therefore lie in facilitating comprehensive staging assessment, multidisciplinary treatment planning, integration of supportive services, optimization of treatment sequencing, and improved communication between specialties. These factors may contribute to more coordinated and patient-centered care, particularly in anatomically and therapeutically complex head and neck malignancies.

MDTs in cancer care consist of healthcare professionals from diverse specialties working together to provide holistic and coordinated care to patients. We found consistent results with previous systematic reviews assessing the fundamental role of MDTs in reducing mortality and enhancing survival. While assessing MDT-related survival among patients with HNC, significantly improved survival was observed in the MDT group [HR = 0.84, 95% CI (0.76–0.92), *P* = 0.0004] compared with the non-MDT group in a report by Shang *et al*^[^22^]^ Also, Shang et al. found that it had a negligible role in stage IV HNC [HR = 0.81, 95% CI (0.59–1.10), *P* = 0.18], and we are in line with their results in stage IV HNC [HR = 0.80, 95% CI (0.62–1.03), *P* = 0.09] as well. Initially, some authors linked a better prognosis with MDT, which was statistically reported to have a limited impact due to higher heterogeneity by Croke et al^[^23^]^ MDT achieved an OS of 83% compared with non-MDT achieving 78% survival, again consistent with our study results^[^14^]^. Statistically speaking [HR = 0.7, 95% CI (0.49–0.99); *P* = 0.03] about reliable results resonating with our findings, survivability was higher among patients with HNC managed under MDT than non-MDT (66.7% vs 46.5%) as reported by Rizky *et al*^[^24^]^.

In agreement with MDT structures for nasopharyngeal carcinoma, improved progression-free survival of up to 59.89 months in MDT patients compared with non-MDT patients (12.68 months) was found to be statistically significant [HR 0.267, 95% CI (0.17–0.40), *P* < 0.001], consistent with our report^[^25^]^. With the rise of HNC, regional sarcomas may complicate management due to variable presentations and radiologic findings, which require MDT. OS of 72% was observed in an MDT study that included 178 patients; in our study, OS was higher by 13% ^[^26^]^. Such a large difference in OS could be due to a greater preponderance of squamous cell carcinoma in our study, which behaves aggressively and carries higher mortality than sarcoma types in the head and neck region, but this hypothesis requires a data-based study^[^27,28^]^.

Taking a different stance from our data, a recent study demonstrated a non-significant effect (*P* = 0.96) of MDT on 5-year survival in either group (MDT and non-MDT), which was hypothesized to be due to advanced-stage cancer in the MDT group and a longer gap between diagnosis and therapy initiation^[^29^]^. However, Friedland *et al* documented a pronounced effect of MDT in stage IV disease rather than stages I–III, providing a broad indication for MDT in managing HNC even at higher stages^[^16^]^.

Some studies have suggested limited implications of MDT beyond stage III, inviting researchers to investigate its significant role via additional studies^[^30^]^. Additionally, our study is in line with these results.

Acceptance of the role of MDT in improving OS has also been observed by many studies when implemented in non-HNCs. An improved OS due to MDT was found to be statistically significant among patients with colorectal cancer [HR = 0.81, 95% CI (0.69–0.94), *P* = 0.005], which statistically correlates with our study on the role of MDT in HNC^[^31^]^. Similarly, MDT played a key role in reducing mortality in the form of reduced recurrence and enhanced survival rates in lung, breast, and prostate^[^32,33^]^. In line with this, the factors involved in such improvement – i.e., stage, age, and timing of diagnosis or therapy–may vary by disease and can be compared with HNC, without the aim of achieving identical goals^[^34^]^.

Discussing complex oncologic involvement of body organs, including the head and neck, within an MDT has been shown to have a direct impact on improved survival rates. Apart from local aggressiveness of disease in the case of HNC, the co-occurrence of variable biological processes may worsen the related OS, which could be alleviated with MDTs^[^35^]^. Optimal decisions, staging with higher accuracy, better treatment adherence, and improved quality of life could be key factors linked with MDTs to improve OS among such patients^[^36^]^.

Preoperative MDT review may cause remarkable changes in individual opinions regarding diagnostic and therapeutic options, as up to 45% of patients attending MDTs received a different management plan toward HNC than before MDTs^[^37^]^. In a similar context, a measurable change in the survival rate of breast cancer patients before (41.7%) and after MDT (56.8%) was observed, encouraging its use in future practice^[^38^]^. A statistically significant impact of MDT [HR = 0.67, 95% CI (0.62–0.71)] was also associated with a survival of 30.2 months, significantly more than the 19.0 months in the non-MDT group, as determined in a systematic review^[^39^]^.

MDTs associated with poor survival outcomes have highlighted serious concerns about the conduct of MDTs by inexperienced personnel, as reported by Van der Vlies *et al*^[^40^]^. Additionally, factors like overburdened caseloads, low interest in MDTs, miscommunication, data compilation errors, poor collaboration among team members, and bias toward a certain plan may adversely affect MDT outcomes, with substantial negative impact, which must be mitigated as soon as possible^[^41^]^.

The substantial heterogeneity observed in the OS analysis should be interpreted in light of the diversity of the included studies. Heterogeneity in meta-analyses may arise from clinical and methodological diversity across included studies. In the present review, such diversity was evident in the study characteristics, including country, study period, sample size, tumor subsite, and clinical setting. The studies were conducted in Taiwan, Australia, the United States, and England, with recruitment periods ranging from 1996 to 2017 and sample sizes ranging from 106 to 19 513 patients. In addition, the included studies evaluated different HNC subsites, including oral cavity and oropharyngeal cancers, as well as broader HNC populations. These differences may partly explain the observed statistical heterogeneity. Although subgroup and sensitivity analyses were performed, the limited number of studies prevented more detailed exploration using meta-regression.

Compared with previous evidence from Shang *et al*^[^22^]^, which suggested a survival benefit of MDT care, our review adds value by including a larger and more recent dataset, broader geographic representation, a subgroup analysis of survival rates for patients with stage IV cancer by type of cancer, and a structured quality assessment of the included studies. These additions provide updated insight into the association between MDT-based care and survival outcomes in patients with HNC.

The findings also indicate the potential disadvantage of non-MDT management in HNC. Non-MDT management has been associated with significantly higher treatment delays, which are particularly concerning in HNC because delayed treatment initiation may result in tumor progression, stage migration, and decreased OS^[^42,43^]^. Furthermore, reduced adherence to evidence-based guidelines in advanced HNC has been associated with poorer survival outcomes^[^44^]^, which may emphasize the importance of coordinated multidisciplinary decision-making. Reviews evaluating MDT implementation in HNC have also demonstrated improved treatment coordination, reduced time-to-treatment, and more consistent guideline-directed care among MDT-managed patients^[^45^]^.

Several limitations of the current study must be acknowledged, which may invite further research opportunities and inform future perspectives. The generalizability of our findings is limited by the geographic distribution of the included studies, which were conducted mainly in Taiwan, the United States, Australia, and England, with a large proportion of the total patient population derived from Taiwanese nationwide datasets. MDT structure, referral pathways, healthcare resources, treatment accessibility, and institutional expertise may differ substantially across regions. Therefore, additional studies from underrepresented populations and healthcare systems are needed to strengthen external validity. The mean ages reported across the included studies ranged from 51 to 70.2 years; patients beyond this age may develop stage IV HNC, for whom applicability regarding OS might be limited. The variable implementation and timing of MDT in our studies may produce variable results, which might be addressed by following international MDT guidelines. Publication bias could not be formally assessed because fewer than ten studies contributed to each meta-analysis; therefore, residual publication or selective reporting bias cannot be excluded. All included studies were retrospective cohort studies; therefore, the certainty of evidence is limited. Retrospective designs may contribute to selection bias, residual confounding, and differences in institutional practice patterns. Patients selected for MDT care may differ from those receiving non-MDT care in terms of disease severity, comorbidities, access to specialized services, socioeconomic status, or treatment adherence. Differences in treatment protocols and MDT implementation may have contributed to variation between studies; however, these factors were not consistently reported in sufficient detail to permit formal subgroup analysis. In addition, variations in MDT organization, specialist availability, meeting frequency, scheduling logistics, and institutional workflow may have influenced the timeliness of treatment initiation across centers. Because these operational factors were inconsistently described in the included studies, their impact on patient outcomes and potential treatment delays could not be quantitatively assessed. Therefore, the observed survival benefit should be interpreted as an association rather than definitive evidence of causality. The quality assessment also identified specific methodological limitations across the included studies. Several studies did not report sample size justification or power calculations, did not assess MDT exposure more than once over time, did not report blinding of outcome assessors, or did not clearly report loss to follow-up. Furthermore, although confounding adjustment was assessed as part of the NIH tool, differences in measured and adjusted confounders across retrospective studies may have affected the estimated association between MDT care and survival. Lastly, conducting MDT is not just a time-consuming task under skilled hands; it may have economic implications that must be assessed during the study. Data depicting the economic burden associated with MDT may improve assessments of its feasibility for the appropriate population.

## Conclusion

This systematic review and meta-analysis suggests that MDT management is associated with improved OS in patients with HNC. However, because all included studies were retrospective and substantial heterogeneity was observed, the findings should be interpreted cautiously. The survival benefit in stage IV disease remains inconclusive due to the small number of studies. Future prospective, multicenter studies should use standardized MDT definitions, report stage-specific outcomes, describe MDT structure and treatment decisions, and adjust for key confounders. Such studies are needed to clarify which HNC patient groups benefit most from MDT care, particularly in advanced-stage disease.

## Data Availability

Data sharing is not applicable to this article as no new datasets were generated or analysed during the current study.

## Research registration unique identifying number (UIN)

This systematic review and meta-analysis was registered in the International Prospective Register of Systematic Reviews (PROSPERO) under the registration number CRD420251165637.
